# Blood Plasma Proteins Associated With Heart Rate Variability in Cosmonauts Who Have Completed Long-Duration Space Missions

**DOI:** 10.3389/fphys.2021.760875

**Published:** 2021-11-17

**Authors:** Ludmila Kh. Pastushkova, Vasily B. Rusanov, Anna G. Goncharova, Andrei M. Nosovskiy, Elena S. Luchitskaya, Daria N. Kashirina, Alexey S. Kononikhin, Anna R. Kussmaul, Yusef D. Yakhya, Irina M. Larina, Evgeny N. Nikolaev

**Affiliations:** ^1^Institute of Biomedical Problems of the Russian Academy of Sciences, Moscow, Russia; ^2^Skolkovo Institute of Science and Technology, Skolkovo, Russia; ^3^V.L. Talrose Institute for Energy Problems of Chemical Physics, N.N. Semenov Federal Center of Chemical Physics, Russian Academy of Sciences, Moscow, Russia

**Keywords:** cosmonauts, long-duration space missions, blood proteome, heart rate variability, sympathetic and, parasympathetic regulation

## Abstract

The study presents the results of evaluating the changes in the concentrations of blood plasma proteins associated with heart rate variability (HRV) in cosmonauts who have completed space missions lasting about 6months. The concentrations of 125 proteins were quantified in biological samples of the cosmonauts’ blood plasma. The subgroups of proteins associated with the physiological processes of the HRV autonomic regulation were identified using bioinformatic resources (Immunoglobulin heavy constant mu, Complement C1q subcomponent subunit C, Plasma serine protease inhibitor, Protein-72kDa type IV collagenase, Fibulin-1, Immunoglobulin lambda constant 3). The concentration of these proteins in the blood plasma before the flight, and the dynamics of concentration changes on the 1st and 7th days of the post-flight rehabilitation period differed in the groups of cosmonauts with a predominance of sympathetic or parasympathetic modulating autonomous influences. The dynamics of changes in the concentrations of the identified set of proteins reveal that in cosmonauts with a predominance of sympathetic modulating influences, the mechanisms of autonomic regulation are exposed to significant stress in the recovery period immediately after the completion of the space mission, compared with the cosmonauts with a predominance of parasympathetic modulating influences.

## Introduction

One of the urgent tasks of space medicine, in our opinion, is the search for biomarkers of cardiac activity before preparation for space flight and at the stage of post-flight rehabilitation. Nowadays the age of cosmonauts is increases, as well as the risk of serious life-threatening cardiovascular events does. The identification of personalized metabolic markers will allow the identification of not present at risk of cardiovascular risks cosmonauts. This is relevant not only for space medicine but also for public health since the number of people, who can move from a group with a low risk of cardiovascular diseases to a group with a high one, when exogenous factors change is quite large. In addition, early diagnostics of the appearance of violations of the mechanisms of regulation of cardiac activity will prevent possible negative effects of lunar soon planned exploration missions and flights beyond the low earth orbit. Thus, need to identify preclinical biomarkers, which characterize the functional state and adaptive capabilities of the body and predict the personalized state of health.

To assess cardiovascular risks in cosmonauts, the Russian space cardiology uses an approach that is primarily based on the study of regulation mechanisms of the circulatory system (Baevsky and Chernikova, 2016).

The analysis of heart rate variability (HRV) is one of the commonly recognized methodological approaches for studying the adaptation to extreme conditions since it allows to assess of the state of systems involved in the regulation of blood circulation (Baevsky and Chernikova, 2017).

The duration of a cardiac cycle can be measured by the RR interval from the electrocardiogram (ECG) because the R peaks are the easiest to detect in the ECG signal. The control of the heart rate is modulated by both sympathetic nervous system (SNS) and parasympathetic nervous system (PNS) branches of autonomic nervous system (ANS). The PNS regulates the hearts’ functions rapidly. In contrast, the SNS regulates the heart functions slower. The ANS is therefore responsible for changing the duration of RR interval from one beat to another (Acharya et al., 2007). This phenomenon is called HRV (Ernst, 2017; Holzman and Bridgett, 2017; Massaro and Pecchia, 2019; Karemaker, 2020).

Despite the available data from the studies of autonomic regulation mechanisms in the cardiovascular system (CVS) conducted on board of the International Space Station (ISS), as well as after space missions (SM; Baevsky et al., 2011, 2013; Hughson, 2012; Vandeput et al., 2013; Xu et al., 2013; Otsuka et al., 2018), the criteria for the probability of adverse cardiovascular events using the assessment of the functional state of cosmonauts according to the HRV analysis and taking into account the identified type of autonomic regulation in space flight conditions have not been defined sufficiently due to the individual typological features of autonomic regulation (Baevsky and Chernikova, 2016).

Proteomic approaches are unequivocally powerful tools that may provide a deeper understanding of the molecular mechanisms associated with cardiovascular events. Cardiovascular proteomics is an emerging field and significant progress has been made during the past few years with the aim of defining novel candidate biomarkers and obtaining insight into molecular physiology and pathophysiology of the cardiovascular system (Mokou et al., 2017).

In our opinion, cardiovascular proteomics reflects the processes of protein interactions in the regulation of the cardiovascular system, and, possibly, the dynamics of adaptation to complex extreme influences, as well as the possibility of returning to the original patterns of the genetically determined regulation of the cardiovascular system. The use of proteomics methods will make it possible to define the participation of the metabolic network in the blood circulation regulation, and, possibly, to identify the metabolic markers involved in maintaining the autonomic homeostasis.

The purpose of the study is to evaluate the changes in the concentrations of blood plasma proteins associated with HRV in cosmonauts who performed long-duration space missions.

## Materials and Methods

The study involved 7 Russian cosmonauts (males, average age 44±6years, body mass index 26.5±2) who performed long-duration space missions on the ISS lasting 169–199days. The investigations were performed on pre-launch days 30–45 (Pre) and on the background of acute readaptation or recovery after landing days 1 (R+1) and 7 (R+1).

All cosmonauts provided written informed consent to participate in the investigations approved by the Biomedicine Ethics Committee of the Institute of Biomedical Problems of the Russian Academy of Sciences at Physiology Section of the Russian Bioethics Committee of Russian Federation National Commission for UNESCO and Human Research Multilateral Review Board, NASA, Houston, TX, United States.

The blood was taken from a vein in the elbow pit 30days prior to the launch and a day after the landing (after 25.2±0.1h) into the SARSTEDT-Monovette^®^ tubes containing EDTA. The plasma was separated by centrifugation and frozen at a temperature of −80°C. No protease inhibitors or antimicrobial agents were added.

The target quantitative analysis was performed using liquid chromatography and tandem mass spectrometry with multiple reactions monitoring (LC/MRM-MS). The LC/MRM-MS analysis was performed on UPLC 1290 Infinity chromatograph system (Agilent Technologies) using a Zorbax Eclipse Plus RP-UHPLC chromatographic column coupled to triple quadrupole mass spectrometer Agilent 6,490 as previously discussed (Larina et al., 2017). MassHunter quantitative analysis software (version B. 07.00, Agilent) was used to analyze LC/MRM data. For target quantitative analysis, BAK 125 kit (MRM Proteomics Inc., Canada) containing both stable-isotope labeled internal standard (SIS) and natural (NAT) synthetic proteotypic peptides was used for concentration measurements of the corresponding 125 proteins in plasma. 13C/15N-labeled peptide analogues were used as internal standards for the quantitative determination of plasma proteins. They were synthesized and purified using reversed-phase high-performance liquid chromatography (RP-HPLC), followed by evaluation on MALDI-TOF-MS. The purity of SIS peptides averaged 94.2% (Kuzyk et al., 2013).

The HRV analysis was carried out in 5-min ECG samples at rest in the supine position. The raw data used for the analysis are presented in the attached file. Cardiovascular regulatory mechanisms condition was assessed according to the recommendations developed by the European cardiological and North American electrophysiological Societies (Camm et al., 1996; Standards of measurement, physiological interpretation, and clinical use, 1996). The HRV analysis was performed at the same time as the collection of samples of biological material.

Depending on the type of autonomic regulation in cosmonauts before the flight, the associative relationships between some proteome proteins and HRV characteristics were analyzed. To determine the molecular functions, biological processes and signaling pathways carried out with the participation of certain proteins, the DAVID online resource^1^ and the PubMed search engine^2^ were used. Additional information about the properties and molecular weight of proteins was obtained using the Uniprot database^3^ and the STRING online software.^4^

The analysis of experimental data was performed using the Factorial ANOVA statistical module of the Statistica v. 7 software package.

Ward’s method of cluster analysis was used for statistical analysis (Hartigan, 1975). The statistical hypothesis that the examined sample was taken from the normal distribution was tested. The statistical Shapiro–Wilk test was used for this purpose. This test is one of the most effective tests of normality, and *p* value was <0.325, i.e., the null hypothesis of belonging to the normal distribution was not rejected. The differences between the experimental samples were found using the Tukey’s honestly significant difference test.

## Results

**Figure 1:**
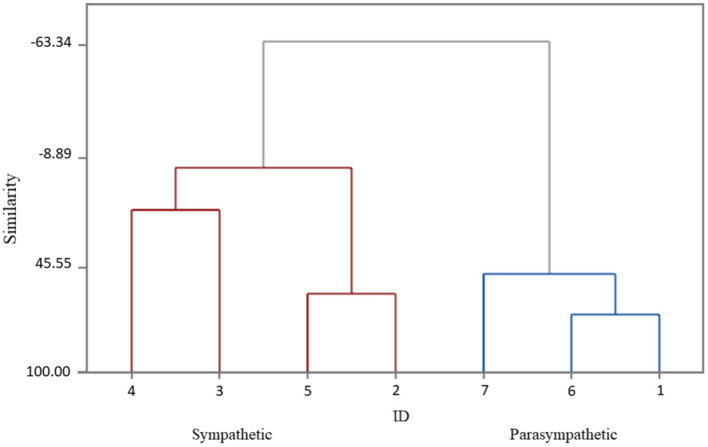
The division of cosmonauts into groups depending on the predominant type of autonomic regulation (Dendrogram Ward Linkage; Euclidean Distance). ID 4; 3; 5; 2 (red line) – cosmonauts with a predominance of sympathetic modulating influences (sympathetic). ID 7; 6; 1 (blue line) – cosmonauts with a predominance of parasympathetic modulating influences (parasympathetic).

We have used Wards’ cluster analysis method, which made it possible, taking into account the individual variance of the initial background indicators, to form stable groups. We classified cosmonauts according to the predominant type of autonomic regulation (sympathetic or parasympathetic) using data of HRV analysis, which allowed, by assessing RR variability, to evaluate the modulating effect of the corresponding section of the ANS on the mechanisms of cardiovascular homeostasis regulation (Figure 1):

Group 1(*n*=4) – cosmonauts with a predominance of sympathetic modulating influences (sympathetic).

Group 2(*n*=3) – cosmonauts with a predominance of parasympathetic modulating influences (parasympathetic).

To obtain the most informative indicators characterizing to the predominant type of autonomic regulation, we used the methods of cluster and discriminant analysis. As a result, the classification functions were determined, which included the following indicators, the most informative for this group of cosmonauts, reflecting autonomic homeostasis: SDNN (ms), pNN50 (%), HF (ms^2^).

SDNN (ms) is the standard deviation obtained for the total dataset of interbeat intervals and reflects the integral effect of regulatory systems.

pNN50 (%) - is the number of pairs of adjacent intervals differing by more than 50ms, in % of the total number of RR intervals in the array, an indicator of the parasympathetic regulatory branch prevalence over the sympathetic.

Power HF (mc^2^) - is the raw power of HRV high-frequency component related to the total power of fluctuations and reflects the relative activity of the parasympathetic regulatory component.

**Figure 2:**
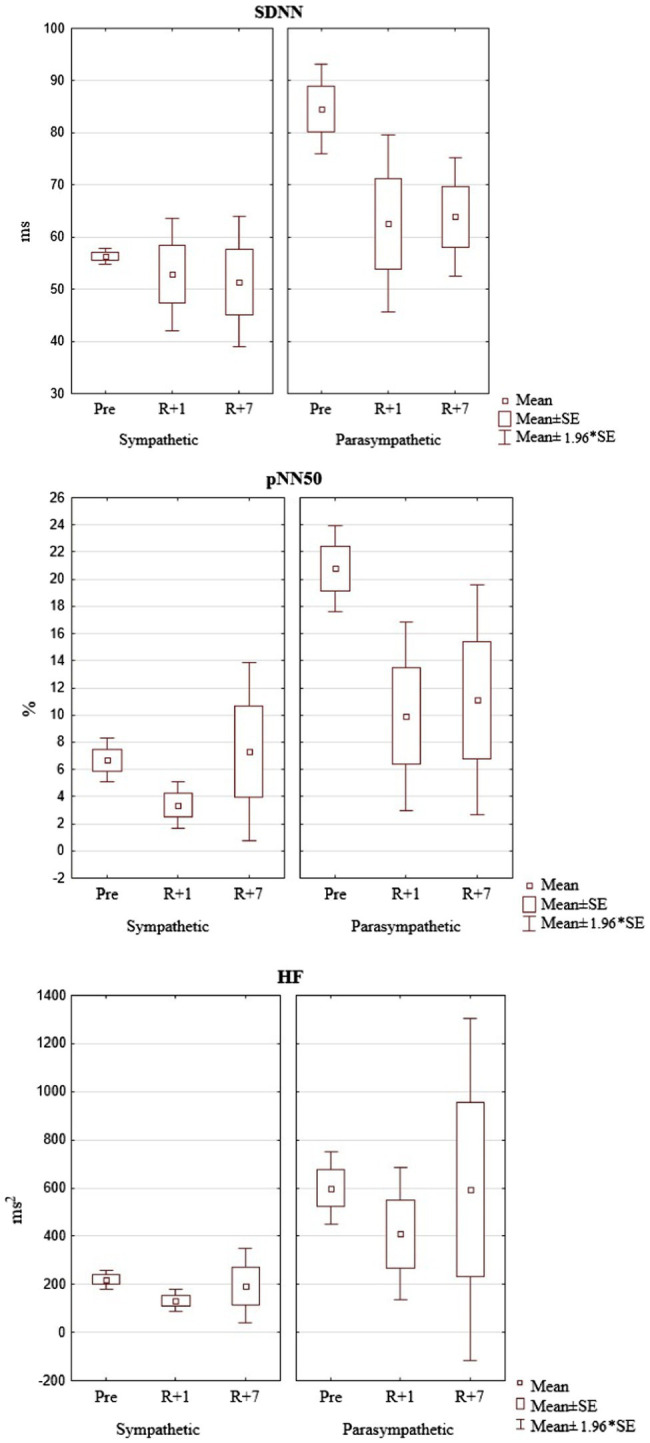
The dynamics of the HRV parameters pre-mission (Pre), on the 1st day (R+1) and on the 7th day (R+7) after landing (the cosmonauts are grouped into clusters depending on the time of research).

Before the mission, these indicators showed significant differences between the groups, which confirmed the dominance of the corresponding types of autonomic regulation (Figure 2).

The influence of factors characteristic for coming back to Earth after a space mission forces the body to re-adapt to earthly conditions providing the necessary cardiovascular homeostasis. At this point, regardless of the regulation type, the maintenance of the adaptive reserves involves the expenditure of a significant amount of energy resources. This is reflected in a decrease in the overall HRV and the effectiveness of regulatory systems (SDNN), as well as a decrease in parasympathetic influences (pNN50 and HF).

Our study is a continuation of previously published data (Larina et al., 2017), which quantified a set of proteins that perform their function in the extracellular fluid and are used clinically to diagnose non-communicable diseases. In conducting our investigation, we focused on those that could be associated with the mechanisms of autonomic regulation of heart rhythm.

The concentrations of 125 proteins have been quantified in the biological samples of the cosmonauts’ blood plasma. After dividing the astronauts into groups depending on the type of autonomic regulation, we determined the statistical differences in the protein concentration before the flight and identified the proteins associated with this physiological process.

Among the 125 proteins, differences in plasma concentration of 6 proteins were noted in the groups of cosmonauts with a predominance of sympathetic or parasympathetic modulating influences before the mission: Immunoglobulin heavy constant mu (gene IGHM); Complement C1q subcomponent subunit C (gene C1QC); Plasma serine protease inhibitor (Gene SERPINA5); Protein-72kDa type IV collagenase (Gene MMP2); Protein-Fibulin-1 (Gene FBLN1); Protein-Immunoglobulin lambda constant 3 (Gene IGLC3).

However, while significant differences in the concentrations of proteins associated with the processes of heart rhythm autonomic regulation were detected before the mission, these features did not occur in the post-mission period (Table 1). Nevertheless, the participation of these proteins in cardiovascular processes may reflect the molecular mechanisms of adaptation in weightlessness depend on different types of autonomic regulation.

**Table 1:** The reliability of differences in the level of proteins between the groups in the baseline period, on the 1st and the 7th days after the mission.

Protein	Value of *P* preflight group 1/ preflight group 2	Value of *P* R+1 group 1/ R+1 group 2	Value of *P* R+7 group 1/ R+7 group 2
Immunoglobulin heavy constant mu	0.016267	0.255209	0.141407
Complement C1q subcomponent subunit C	0.022965	0.687916	0.439854
Plasma serine protease inhibitor	0.009249	0.507887	0.471242
Protein-72kDa type IV collagenase	0.041004	0.134357	0.302210
Fibulin-1	0.000652	0.488081	0.837809
Immunoglobulin lambda constant 3	0.010391	0.689207	0.663312

**Figure 3:**
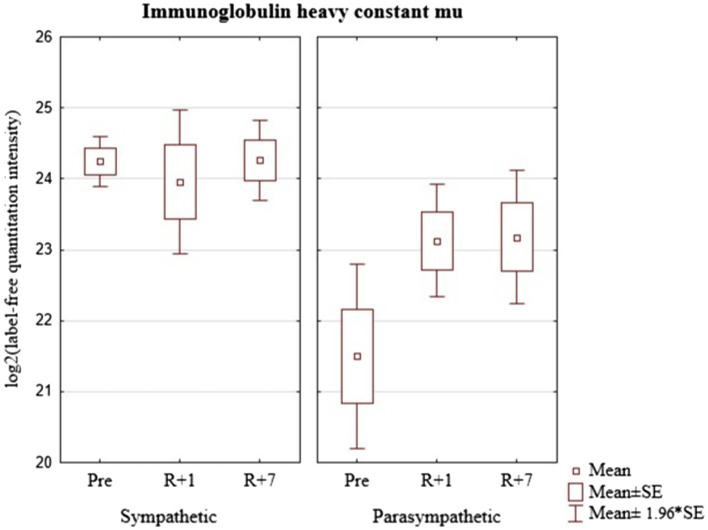
Immunoglobulin heavy constant mu (gene IGHM) in cosmonaut groups pre-mission (Pre), on the 1st day (R+1) and on the 7th day (R+7) after landing.

There were no significant changes in the level of IGHM after the space mission in the group of cosmonauts with a predominance of sympathetic modulating influences. The group of cosmonauts with a predominance of parasympathetic modulating influences demonstrated a moderate increase in the content of this protein that persisted to the 7th day after landing. Thus, the discovered baseline differences in individuals with a predominance of sympathetic or parasympathetic regulatory influences became less noticeable in the post-mission period. At the same time, the dynamic of IGHM concentration in the group with a predominance of parasympathetic modulating influences may indicate that the cardiovascular system of the cosmonauts with this type of autonomic influences is more exposed to space flight factors (Figure 3).

**Figure 4:**
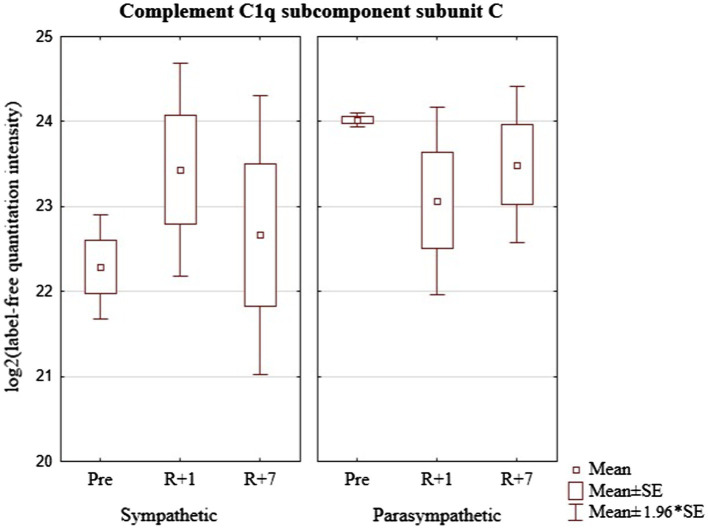
Complement C1q subcomponent subunit C (geneC1q) in cosmonaut groups pre-mission (Pre), on the 1st day (R+1) and on the 7th day (R+7) after landing.

The data presented in Figure 4 show that before the mission the concentration of C1QC significantly differs in cosmonauts with a predominance of sympathetic or parasympathetic influences. However, on the first day after landing and seven days after the end of the mission, the groups do not differ. The group with a predominance of sympathetic modulating influences revealed an increase in the level the protein on the first day with a tendency to return to the baseline values on the seventh day after landing. On the contrary, there was a tendency to decrease on the first day after the flight with the return to baseline values on the seventh day in the group with a predominance of parasympathetic modulating influences. These multidirectional trends lead to smoothing out the differences noted in the baseline period.

**Figure 5:**
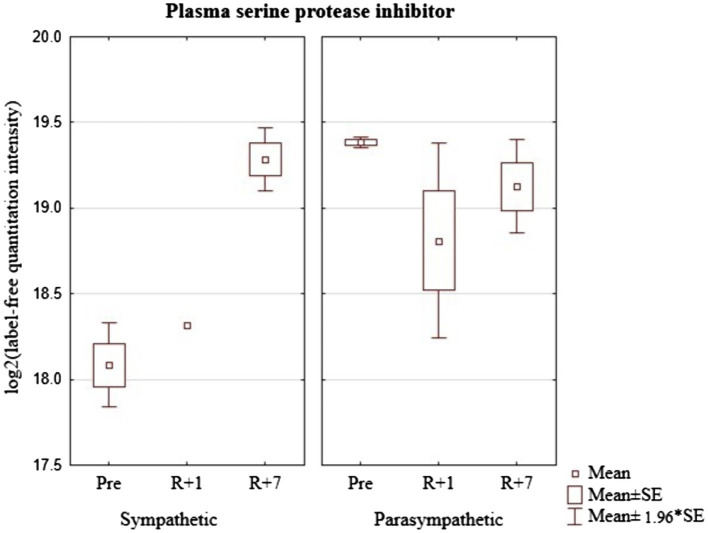
Plasma serine protease inhibitor (Gene-SERPINA5) in cosmonaut groups pre-mission (Pre), on the 1st day (R+1) and on the 7th day (R+7) after landing.

Similar to the previous protein markers, the level of SERPINA5 significantly differed in the identified groups before the mission. The effect of space flight on its concentration in the blood was different in the two selected groups (Figure 5). In addition, in the group with a predominance of sympathetic modulating influences, there was a tendency to increase the level of plasma serine protease inhibitor on the seventh day after landing. In the second group, on the contrary, there was a tendency to decrease on the first day after the flight with a relative increase to the level of baseline values on the seventh day. Thus, the space flight conditions have opposite effects on the level of plasma serine protease inhibitor in the groups of cosmonauts with different dominant autonomic influences.

**Figure 6:**
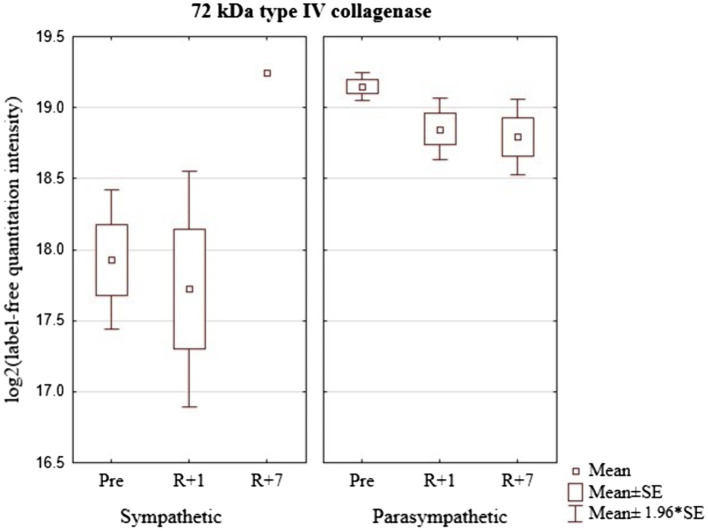
Protein-72kDa type IV collagenase (Gene MMP2) in cosmonaut groups pre-mission (Pre), on the 1st day (R+1) and on the 7th day (R+7) after landing.

As can be seen from Figure 6, the group of cosmonauts with a predominance of sympathetic modulating influences showed no changes in the level 72kDa type IV collagenase on the first day after the mission, but an increase in the level of this protein was noted on the seventh day of the recovery period. On the contrary, the group of cosmonauts with a predominance of parasympathetic modulating influences demonstrated a tendency to decrease the level of this protein on the first day and the seventh day after the mission.

**Figure 7:**
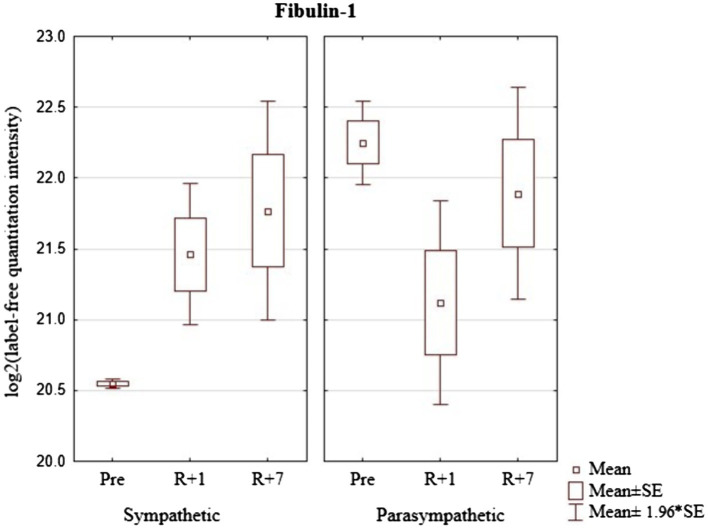
Protein-Fibulin-1 (Gene FBLN1) in cosmonaut groups pre-mission (Pre), on the 1st day (R+1) and on the 7th day (R+7) after landing.

The group of cosmonauts with a predominance of sympathetic modulating influences demonstrated a pronounced increase in the level of FBLN1on the first day with a continued increase by the seventh day of the recovery period (Figure 7). Cosmonauts with a predominance of parasympathetic modulating influences, on the contrary, showed a tendency to a sharp decrease in its level on the first day and a relative increase, almost to baseline values on the seventh day after the mission.

**Figure 8:**
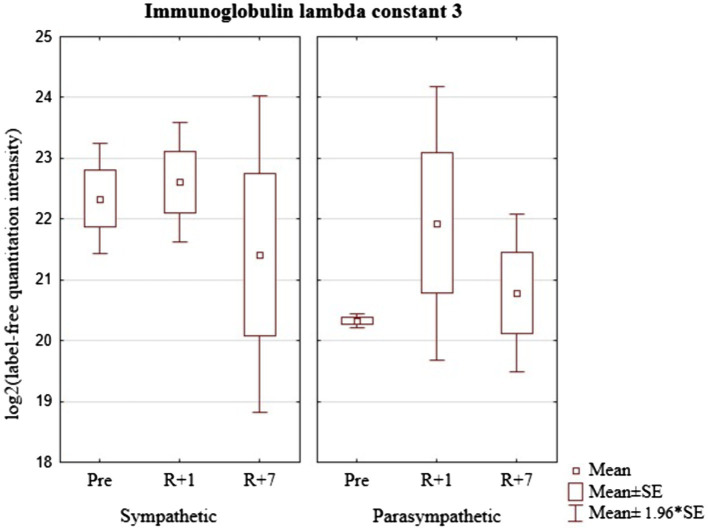
Immunoglobulin lambda constant 3 (Gene IGLC3) levels in cosmonaut groups pre-mission (Pre), on the 1st day (R+1) and on the 7th day (R+7) after landing.

In the group of cosmonauts with a predominance of sympathetic modulating influences, there were no changes in the level of IGLC3 after the mission. The group of cosmonauts with a predominance of parasympathetic modulating influences indicated a tendency to increase on the first day and a relative decrease on the seventh day after the mission. In addition, the concentration of this protein was significantly higher in cosmonauts with a predominance of sympathetic modulating influences (Figure 8).

## Discussion

The significant intergroup differences discovered during the statistical analysis were expressed before the space mission. Therefore, the cosmonauts with a predominance of sympathetic and parasympathetic types of autonomic regulation were unequally exposed to space flight factors. However, after the mission the differences in the levels of the identified proteins between the groups were leveled, there were no significant differences between the group’s average indicators either on the 1st or 7th day after landing.

On one hand the data obtained can obviously indicate that the extreme factors identical in duration and direction of exposure have such an intense effect on the body that the final molecular parameters become unified in the processes ensuring an adequate level of cardiovascular system functioning under the changed conditions, regardless of the differences in HRV regulation.

On the other hand, the intra-group dynamics of changes in the concentrations of the discussed set of proteins compared to the preflight values shows that the mechanisms of autonomic regulation in cosmonauts with a predominance of sympathetic modulating influences are significantly more exposed to stress under the influence of space flight factors (and its final stage) than in cosmonauts with a predominance of parasympathetic modulating influences. In the first group, among the proteins associated with HRV regulation, the majority of proteins increased their levels by the 1st and/or 7th days after the mission. On the contrary, the concentration of most of these proteins in the second group decreased. It is obvious that the limiting factor of our research is the insignificant number of cosmonauts who took part in it, and our assumptions, undoubtedly, should be confirmed or refuted on more representative cosmonaut groups.

Using bioinformatic resources, we focused on the biological functions of the identified proteins and protein interactions that may be reflected in the mechanisms of the heart rate autonomic regulation.

### Immunoglobulin Heavy Constant Mu (gene IGHM)

It is known that the IgM level correlates with higher systolic blood pressure and may indirectly reflect the phenomena of subclinical peripheral atherosclerosis (Swärd et al., 2020). On the other hand, the effect of space flight on the dynamics of the range of antibodies in a healthy person has not been sufficiently studied. When studying the IgM of five cosmonauts 25days prior to the launch, then after 64±11 and 129±20days spent on the ISS, and then 1, 7 and 30days after the landing, it was found that the IgM representation in the cosmonauts differs from the control samples (*n*=4) before the launch, and that the samples of two of the five analysed cosmonauts showed significant changes in the IgM spectrum during the mission. These modifications were noticeable for a period of up to 30days after the landing. These changes affected the specificity of IgM binding sites, and coincided with a higher response to stress, which is confirmed by the data we obtained on the relationship of IgM changes and the implementation of an individual response to stress (Buchheim et al., 2020; Bajwa and Mohammed, 2021).

### Complement C1q Subcomponent Subunit C (gene C1qc)

C1q is a target recognition protein of the classical complement pathway. It is believed that the components of the C1qc complement are responsible for “aging,” myocardial fibrosis (Bartling et al., 2019). Considering the connection of C1q with an individual response to stress, it is necessary to note the proven connection between a number of protein components, including Hspd1, Actb, Mgst1, THBS4, Syp, C1q, Serpine, Plat and Ngf, which are associated with cellular stress, neural plasticity reactions and hippocampal responses to trauma and damage (Hao and Wang, 2017).

### Plasma Serine Protease Inhibitor (Gene SERPINA5)

In regard to our study, the role of SERPINA5—an inhibitor of plasma serine protease—is expressed in its participation in the regulation of intravascular and extravascular proteolytic activity that ensures coagulation (thrombosis and thrombolysis), neurotrophic effects, hormone transportation, the activation of the complement system, pro-inflammatory activity and angiogenesis (Meijers et al., 2002). It is noted that SERPINA5 indirectly regulates blood pressure, HRV and numerous other physiological processes (Koukos et al., 2011; Zheng et al., 2013). This protein is an inhibitor of plasma serine protease and a powerful inhibitor of activated protein C (APC), which plays an important role in the pathway of anticoagulant protein C and at tissue damage sites in tissue regeneration processes (Hughson, 2012).

### Protein-72kDa Type IV Collagenase (Gene MMP2)

From the point of view of the main processes that distinguish the groups of cosmonauts with different types of heart rhythm autonomic regulation, MMP2-72kDa type IV collagenase provides the destruction of extracellular matrix proteins and impacts several non-matrix proteins that stimulate vasoconstriction (Chan et al., 2019). It also participates in the formation of fibrovascular tissues in association with MMP-14, as well as the in vascular network remodelling, angiogenesis, tissue repair, inflammation and rupture of atherosclerotic plaques (Batista et al., 2019).

The myocardial expression of MMP-2 has been noted to increase with heart failure and pressure overload (Barhoumi et al., 2017). By affecting the formation of endothelin-1, MMP-2 causes vasoconstriction thus regulating vascular tone and reactivity.

There is evidence that chronic psychological stress is associated with increased expression and activity of mRNA matrix metalloproteinases MMP-2 and MMP-9, as well as the destruction of elastin in damaged carotid arteries (Meng et al., 2020).

### Protein-Fibulin-1 (Gene FBLN1)

FBLN1 is a member of the extracellular matrix glycoprotein family, involved in such cellular functions as adhesion, migration and differentiation and fibrosis. Fibulin can also play a role in hemostasis and thrombosis due to its ability to bind fibrinogen and to be included in the blood clot (Sang et al., 2021).

FBLN1 together with MMP2 are involved in the organization of the extracellular matrix and the change of its properties.

The analysis of hereditary disorders associated with altered collagen structure or leading to its excessive degradation allows to make a conclusion on the functional significance of collagen as an ECM element for assessing the state of the vascular wall, that is reflected in the autonomic regulation of heart rhythm (Arseni et al., 2018).

### Protein- Immunoglobulin Lambda Constant 3 (Gene IGLC3)

The secreted immunoglobulins mediate the effector phase of humoral immunity, which results in the elimination of bound antigens (Schroeder and Cavacini, 2010; McHeyzer-Williams et al., 2012). The role of immunoglobulin (Protein - Immunoglobulin lambda constant 3) in the aspect of its effect on HRV has not been sufficiently covered in the available literature. It is possible that its participation in the cardiovascular system functions is not direct but mediated by other physiological processes that respond to space flight effectors. In our study, it was noted as a protein with significantly different concentrations between groups of cosmonauts with a predominance of sympathetic or parasympathetic modulating influences.

In previous studies of the cosmonauts’ urine proteome, we identified various groups of proteins associated with heart rhythm regulation (Pastushkova et al., 2019, 2020). It was shown that the urine proteome in individuals with the predominance of sympathetic and parasympathetic regulation differed in three proteins: cadherin-13, mucin-1, alpha-1 of collagen subunit type VI, which does not contradict, but complements the results of the blood proteome described above. It should be noted that in this work, the list of quantifiable proteins was narrower, since it was studied on a targeted basis, and was based on the results of previous studies that allowed us to narrow the search patterns.

Using the STRING software, we identified the processes that connect the abovementioned proteins of the blood proteome and the previously studied urine proteins. It is noteworthy that both these blood proteins and urine proteins are involved in the implementation of the same biological processes (Table 2).

**Table 2:** Characteristics of the main processes regulating HRV common to the blood and urine proteome in the baseline period.

Search ID	Process	Number of participating proteins	Names of participating proteins
GO:0007162	negative regulation of cell adhesion	3	CDH13,FBLN1,MUC1
GO:0032101	regulation of response to external stimulus	4	MMP2,CDH13,C1QC,MUC1
GO:0048661	positive regulation of smooth muscle cell proliferation	2	MMP2,CDH13
GO:0072376	protein activation cascade	2	FBLN1,C1QC
CL:731	Collagen formation, and Molecules associated with elastic fibers	3	MMP2,COL6A5,FBLN1
CL:732	Collagen biosynthesis and modifying enzymes, and Elastic fiber formation	2	COL6A5,FBLN1
hsa04610	Complement and coagulation cascades	2	SERPINA5,C1QC

## Conclusion

Undoubtedly, the controlling mechanisms of the ANS reflected in the HRV are genetically determined, but they undergo changes in healthy individuals with age, as well as under the influence of extreme environmental factors.

In weightlessness, the new hemodynamic situation causes changes in the functioning of the mechanisms of blood circulation autonomic regulation. At the same time, the blood proteome dynamically reacts to a set of external and internal space flight factors, ensuring the adaptation of the cosmonauts’ bodies.

The changes in modulating regulatory influences reflected in the HRV are aimed at maintaining the autonomic homeostasis. In our study, this is confirmed by the fact that before the space mission, specific features of both HRV and the concentration of certain proteins of the blood proteome were noted in cosmonauts with a predominance of sympathetic or parasympathetic influences. Staying in the conditions of a long-duration space flight levels the types of regulation and changes the proteomic composition. On the first day after landing, a new HRV-associated proteomic composition is formed that probably ensures the success of the acute period of readaptation after landing, while dynamically changing by the seventh day. The intra-group dynamics of the changes in the concentrations of the discussed set of proteins show that the mechanisms of HRV regulation in cosmonauts with a predominance of sympathetic modulating influences are more exposed to stress than same in astronauts with a predomination of parasympathetic modulating influences in the readaptation period after the space mission in comparison with the average group concentrations before the mission.

## Data Availability Statement

The raw data supporting the conclusions of this article will be made available by the authors, without undue reservation.

## Ethics Statement

The studies involving human participants were reviewed and approved by Biomedicine Ethics Committee of the Institute of Biomedical Problems of the Russian Academy of Sciences at Physiology Section of the Russian Bioethics Committee of Russian Federation National Commission for UNESCO and Human Research Multilateral Review Board, NASA, Houston, TX, United States. The patients/participants provided their written informed consent to participate in this study.

## Author Contributions

IL, VR, EN, and EL conceived and designed the experiments. LP, VBR, DK, and EL performed the experiments. AK, AG, AN, and YY analyzed the data. VR, LP, AG, and AK wrote the paper. All authors read and approved the final manuscript.

## Funding

The study was supported by Russian Science Foundation, grant #19-14-00306 (in part of quantitative blood proteomic study by mass spectrometry). The work was carried out within the framework of the basic Russian Academy of Sciences themes 64.1 and 65.3 for 2013–2023 years.

## Conflict of Interest

The authors declare that the research was conducted in the absence of any commercial or financial relationships that could be construed as a potential conflict of interest.

## Publisher’s Note

All claims expressed in this article are solely those of the authors and do not necessarily represent those of their affiliated organizations, or those of the publisher, the editors and the reviewers. Any product that may be evaluated in this article, or claim that may be made by its manufacturer, is not guaranteed or endorsed by the publisher.

## Footnotes

^1^ https://www.david.ncifcrf.gov

^2^ https://www.ncbi.nlm.nih.gov/pubmed

^3^ https://www.uniprot.org

^4^ https://www.string-db.org
